# Hypothalamic Microglia as Dual Hubs Orchestrating Local and Systemic Homeostasis in the Periphery–Central–Periphery Axis

**DOI:** 10.3390/cells14221780

**Published:** 2025-11-13

**Authors:** Yuan Liu, Qian Jiang, Yimin Huang, Xincheng Zhang, Huayu Kang, Chenxuan Yu, Yuze Xia, Yanchao Liu, Huaqiu Zhang

**Affiliations:** 1 Department of Neurosurgery, Tongji Hospital of Tongji Medical College of Huazhong University of Science and Technology, Wuhan 430030, China; 2 Hubei Key Laboratory of Neural Injury and Functional Reconstruction, Huazhong University of Science and Technology, Wuhan 430030, China

**Keywords:** microglia, hypothalamus, neuroendocrine, neuroimmunity, hypothalamus-periphery direct remote control

## Abstract

**Highlights:**

**What are the main findings?**

- Microglial Role in Homeostasis: The article emphasizes that hypothalamic microglia play a crucial role in regulating both local and systemic homeostasis, acting as key mediators between the central nervous system and peripheral systems.
- Pathological Responses: It highlights that under pathological conditions, microglia can modulate inflammatory responses and initiate repair mechanisms, which are vital for maintaining homeostasis during disease.

**What are the implications of the main findings?**

- Neuroimmune Interaction: The findings suggest that understanding microglia’s role in neuroimmune interactions could lead to new therapeutic strategies for conditions involving dysregulation of homeostasis, such as metabolic disorders and chronic inflammation.
- Potential for Targeted Treatments: By targeting microglial pathways, there is potential for developing treatments that could restore balance in neuroendocrine and immune functions, enhancing recovery from various diseases.

**Abstract:**

The hypothalamus orchestrates systemic homeostasis by integrating neuroendocrine, autonomic, and immune–metabolic functions. In this review, we synthesize evidence that hypothalamic microglia act as dual hubs along a periphery–central–periphery axis across three domains: Peripheral effects: Microglia decode circulating cues and relay them to hypothalamic circuits, thereby modulating autonomic tone and endocrine pathways that impact immune–metabolic balance. Central mechanisms: Microglia sense and shape hypothalamic neural circuits via cytokine–neurotransmitter crosstalk, synaptic remodeling, and glial–neuronal signaling that tune neuroendocrine output. Translational links: Microglial states associate with biomarkers and clinical phenotypes in metabolic and inflammatory disorders, suggesting therapeutic entry points. Drawing on the literature from the last 20 years, we highlight convergent mechanisms, unresolved heterogeneity across models, and priorities for causal dissection and target validation. This framework clarifies how hypothalamic microglia coordinate local CNS processes with systemic physiology in health and disease.

## 1. Background

The hypothalamus, an important component of the diencephalon, is anatomically located beneath the thalamus, surrounding the third ventricle. Its superior portion adjoins the thalamus, while the inferior extension connects to the pituitary gland. The anterior boundary is demarcated by the lamina terminalis superior to the optic chiasm, with the posterior limit continuous with the mammillary bodies of the midbrain in humans [1,2,3]. Despite constituting only 0.3% of total brain weight, the hypothalamus exhibits a complex distribution of nuclei [4,5,6]. Anatomically, this structure can be divided into three principal regions: the anterior region containing the supraoptic and paraventricular nuclei, the middle region housing the ventromedial and dorsomedial nuclei, and the posterior region comprising the mammillary and posterior hypothalamic nuclei. Additionally, the lateral hypothalamic area encompasses various functionally distinct nuclei [7,8,9,10,11,12]. The hypothalamic nuclei integrate neural information through several fundamental pathways that collectively regulate essential physiological processes. As the principal neuroendocrine control center, the hypothalamus orchestrates pituitary function through the hypothalamic-pituitary axis by secreting releasing hormones including corticotropin-releasing hormone (CRH), thyrotropin-releasing hormone (TRH), and gonadotropin-releasing hormone (GnRH) [13,14,15,16]. This neuroendocrine regulation extends to direct secretion of neuropeptides such as antidiuretic hormone and oxytocin, which modulate diverse physiological functions ranging from fluid homeostasis to reproductive behaviors [17,18,19]. Beyond endocrine control, the hypothalamus serves as the supreme autonomic regulator, maintaining internal homeostasis through balanced modulation of sympathetic and parasympathetic outflow. This autonomic integration governs core physiological parameters, including thermoregulation, metabolic balance, and cardiovascular function [20]. The hypothalamus further coordinates higher neural functions through extensive connections with adjacent nuclei, influencing circadian rhythms, emotional states, and cognitive processes [21,22,23,24]. Furthermore, the hypothalamus exerts regulatory control over higher-order neural functions through its extensive interconnectivity with adjacent neural nuclei (network of adjacent neural nuclei). This sophisticated neural network enables the hypothalamus to modulate critical processes, including circadian rhythm synchronization, affective state regulation, and memory consolidation [25,26,27,28]. Contemporary research has significantly expanded our understanding of hypothalamic function, revealing its role not merely as an integrative center but as a pivotal node in complex neural feedback circuits. A growing body of research highlights the hypothalamus’s involvement in sophisticated “body-brain-body” regulatory axes that extend beyond traditional homeostatic functions to encompass various pathological processes. These advances underscore the hypothalamus’s central position in both physiological regulation and disease mechanisms [29,30,31]. Emerging evidence has transformed our conceptualization of the hypothalamus from a traditional neuroendocrine center to a sophisticated neurointegration hub that orchestrates multi-system interactions [32,33,34]. Within this paradigm, microglia—the resident immune cells of the central nervous system—have been identified as key mediators in both physiological maintenance and pathological disruption of hypothalamic neural circuits. These immune cells participate in diverse regulatory processes, including those previously described as core hypothalamic functions [35,36,37].

Studies show that microglia are myeloid progenitor cells originating from the yolk sac, migrating into the brain during embryonic development and maintaining self-proliferation in the nervous system [38,39,40]. Peripheral circulating signals gain access to the brain through circumventricular organs, specialized structures characterized by fenestrated vasculature that lack the protective blood–brain barrier (BBB). Microglia, by virtue of their inherent plasticity and strategic positioning at the vascular-neuronal interface, plays a pivotal role in integrating and relaying peripheral information to neuronal networks. This microglia-mediated signaling pathway constitutes a critical mechanism for the neuroendocrine regulation of systemic metabolism [41,42]. Similarly to peripheral macrophages, they exhibit a branched morphology at homeostatic condition but quickly transform into an amoeboid shape upon reactivation by various stimuli to perform immune functions [43]. Hypothalamic microglia exhibit region-specific functional properties that are critical for both physiological homeostasis and pathological processes. In the homeostatic state, these specialized immune cells maintain neural circuit plasticity through continuous surveillance and activity-dependent synaptic pruning, thereby modulating essential hypothalamic functions including energy balance, thermoregulation, and circadian rhythms [44]. Their homeostatic regulation involves a delicate balance between pro-inflammatory and anti-inflammatory cytokine release, coupled with the ability to sense and respond to metabolic hormones such as leptin and insulin [45,46]. However, under pathological conditions, microglia undergo maladaptive activation characterized by excessive neuroinflammation, disrupted synaptic remodeling, and aberrant phagocytic activity. These pathological transformations ultimately contribute to the development of various metabolic and neurological disorders associated with hypothalamic dysfunction [47,48].

The hypothalamic microglial population is traditionally maintained through local proliferation under physiological conditions [49,50,51]. Emerging evidence, however, reveals a more complex replenishment mechanism during pathological states, where circulating bone marrow-derived monocytes may be recruited to the hypothalamus and differentiate into microglia-like cells. (Traditionally, it is believed that microglia in the adult brain form a self-sustaining population, which is replenished solely through local proliferation. However, emerging evidence indicates that under severe or chronic pathological conditions, the brain’s immune-privileged status is disrupted. At this point, bone marrow-derived monocytes in the circulatory system can be recruited into the brain and differentiate into cells that are similar to microglia in terms of morphology and function. Nevertheless, these cells differ from genuine microglia in terms of origin and nature. They are not the “orthodox” microglia that originate from the yolk sac during the embryonic stage and persist in the brain throughout life. Instead, they derive from hematopoietic stem cells in the bone marrow of adults and are monocytes in the circulatory system. When significant pathological changes occur in the brain (such as chronic neuroinflammation, neurodegenerative diseases, or disruption of the blood–brain barrier), a large number of chemokines are produced. These signals “attract” monocytes in the blood to cross the damaged or more permeable blood–brain barrier and enter the brain parenchyma. After entering the brain tissue, under the influence of the local microenvironment, these monocytes undergo changes in their morphology and gene expression profiles, becoming highly similar to resident microglia—their cell bodies shrink and they extend protrusions. Hence, they are termed “microglia-like cells”.) [52,53,54]. This peripheral infiltration contributes significantly to neuroinflammatory responses in disease conditions. For example, in hypertension, hemodynamic alterations trigger the paraventricular nucleus (PVN) vascular regulatory system to recruit extraneous microglial precursors. Similarly, chronic stress induces PVN neurons to overexpress monocyte chemoattractant protein-1 (MCP-1), facilitating the accumulation and activation of bone marrow-derived macrophages [55].

To delineate the major themes, we divided the review into three parts. Part I examines hypothalamic microglial activation by afferent stimuli along the periphery–brain axis. Part II explores mechanistic insights into microglia as central hubs of hypothalamic neuro–immune–endocrine–metabolic integration. Part III discusses targets and regulatory functions of hypothalamic microglia as orchestrators of the center–periphery axis. The manuscript is based on peer-reviewed literature published over the last 20 years focused on hypothalamic microglia and their roles in neuroendocrine, autonomic, and immune–metabolic regulation; non-peer-reviewed reports and purely non-hypothalamic studies were excluded unless mechanistically informative.

## 2. Hypothalamic Microglia Activation by Afferent Stimuli via the Periphery–Brain Axis

**Figure 1:**
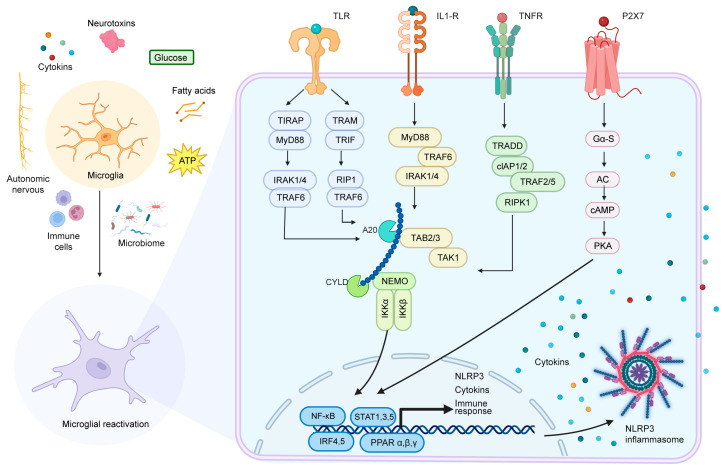
Microglial reactivation: communication pathways between peripheral organs and the hypothalamus. Peripheral tissues and organs activate hypothalamic microglia through various mechanisms, including the release of inflammatory factors, mobilization of immune cells, alterations in gut microbiota, activation of the autonomic nervous system, secretion of metabolic byproducts and neurotoxic substances, etc. These pathways engage receptors such as TLRs, IL-1R, and TNFR, leading to the reactivation of microglia. This reactivation results in increased expression of the NLRP3 inflammasome, cytokines, and proteins related to immune responses, ultimately affecting other hypothalamic cells and facilitating corresponding regulatory responses.

The hypothalamus functions as a master regulatory center that orchestrates whole-body homeostasis by integrating diverse metabolic and immune signals from peripheral organs, particularly adipose tissue, liver, and gut. This bidirectional communication enables precise modulation of energy balance, neuroendocrine activity, and immune homeostasis. Hypothalamic microglia serve as specialized immune sentinels in this network, equipped with an array of detection systems that include pattern recognition receptors (PRRs), such as Toll-like receptors and NLRP3 inflammasomes, and cytokine receptors like IL-1R and TNFR [56]. These immune surveillance mechanisms operate in concert with metabolic sensing pathways mediated by free fatty acid receptors and insulin receptors. Through this integrated signaling apparatus, microglia transduce peripheral information to hypothalamic neurons, ultimately shaping neuronal excitability and functional connectivity within neural circuits governing systemic physiology (Figure 1, Table 1).

Peripheral signals that trigger hypothalamic microglia activation are highly diverse, with lipid metabolites being one of the most well-characterized and physiologically relevant stimuli—especially in the context of diet-induced metabolic dysfunction. As the primary end products of peripheral lipid metabolism, saturated fatty acids, lipopolysaccharides (a byproduct of gut dysbiosis often associated with lipid overload), and other lipid derivatives can readily reach the hypothalamus through specialized anatomical structures, thereby initiating microglial activation cascades. The following section focuses on how these lipid metabolism-related products interact with hypothalamic microglia and the downstream molecular pathways that drive neuroinflammation and metabolic imbalance.

### 2.1. Microglia Activation by Lipid Metabolism-Related Products

Extensive research has established that high-fat diet (HFD) consumption induces hypothalamic neuroinflammation through multiple interconnected pathways, ultimately disrupting metabolic homeostasis [57,58]. The circumventricular organs (CVOs), including regions like the hypothalamic median eminence (ME), are inherently leaky despite being adjacent to areas possessing a sealed blood–brain barrier. CVO neurons detect signaling compounds and secrete hormones into circulation, facilitating rapid communication with the periphery to regulate processes such as feeding, cardiovascular function, and thirst. Microglia in the hypothalamic region densely populate the ME, encasing fenestrated portal capillaries and hypothalamic vascular networks. Microglia within these regions serve as critical communication bridges, establishing direct contact with local capillaries due to the permeable vasculature [59,93,94]. Under conditions of HFD consumption, elevated circulating levels of saturated fatty acids, like palmitate (PA) and lipopolysaccharides reactive microglial TLR4 signaling and downstream NF-κB pathways, promoting excessive production of pro-inflammatory cytokines [60,61,62,95]. Furthermore, certain free fatty acids can directly interact with microglia; binding to TLR2/4 receptors activates the NLRP3 inflammasome, leading to increased IL-1β release and subsequent neuronal apoptosis [63]. Microglia propagate local inflammation via IKKβ/NF-κB signaling cascades, impairing the function of glucose-sensing neurons and exacerbating metabolic dysregulation [96]. These inflammatory mediators exert profound metabolic effects, exemplified by TNF-α, which specifically impairs insulin signaling in pro-opiomelanocortin (POMC) neurons by suppressing IRS-1 tyrosine phosphorylation, contributing to leptin resistance and hyperphagia [65,66,97]. HFD-induced microglial reactivation also promotes aberrant synaptic remodeling, characterized by excessive pruning of inhibitory synapses on agouti-related peptide (AgRP) neurons, thereby disrupting normal energy balance regulation [64]. Concurrently, IL-6 secretion by HFD-activated microglia exhibits dual effects: it stimulates the proliferation of neural precursor cells in the hypothalamic ventricular zone (HVZ) via the STAT3 signaling pathway, while simultaneously exacerbating inflammatory damage within POMC neurons [67,98]. Consequently, diverse pathophysiological responses affecting circulating lipid metabolites and their derivatives can reactive hypothalamic microglia through these interconnected signaling pathways, ultimately exerting significant downstream effects.

While lipid metabolites act as “metabolic stress signals” to activate hypothalamic microglia, peripheral hormones—key regulators of systemic homeostasis—provide another critical layer of microglial modulation. Unlike lipid metabolites that primarily reflect nutrient status, hormones such as leptin and glucocorticoids convey more specific physiological states, enabling microglia to adjust their function in response to changes in energy storage or stress levels. Notably, these hormonal signals often interact with lipid-induced pathways to fine-tune microglial activity, and their dysregulation in pathological states, such as obesity-related hyperleptinemia, chronic stress-induced glucocorticoid excess, can trigger maladaptive microglial activation. The following section explores how leptin and glucocorticoids modulate hypothalamic microglia and the implications of this crosstalk for metabolic and neuroendocrine homeostasis.

### 2.2. Microglia Modulation by Hormonal Signaling

Leptin, an adipocyte-derived hormone, plays a pivotal role in regulating energy homeostasis by modulating food intake, energy expenditure, and metabolic processes through its actions on hypothalamic neurons [74,75,99]. Beyond its neuronal effects, leptin directly modulates microglial function via the long-form leptin receptor (LepRb), triggering downstream signaling cascades including JAK2/STAT3, PI3K/Akt, and ERK1/2 pathways [70]. In obese states characterized by hyperleptinemia, chronic leptin exposure induces LepRb desensitization via the JAK2/STAT3-SOCS3 feedback loop while paradoxically activating pro-inflammatory NF-κB and NLRP3 inflammasome pathways [76]. This pathological switch transforms microglia into a pro-inflammatory phenotype with excessive cytokine release. The resulting impairment of leptin signaling in the arcuate nucleus (ARC) neurons disrupts satiety signaling, while microglia-mediated downregulation of neuronal insulin receptors through IKKβ/NF-κB signaling further exacerbates metabolic dysfunction [71,100]. Notably, microglia promote inhibitory serine phosphorylation of IRS-1 rather than the normal tyrosine phosphorylation, thereby blocking PI3K/Akt signaling and amplifying systemic insulin resistance [70,77,78,101]. This multifaceted dysregulation creates a self-perpetuating cycle: leptin resistance promotes neuroinflammation, which in turn exacerbates both leptin and insulin resistance through synaptic pruning abnormalities and neural circuit disruption. The convergence of these pathological mechanisms underscores the complex interplay between metabolic dysfunction and neuroinflammation in the hypothalamus.

Glucocorticoids exert significant modulatory effects on hypothalamic microglial reactivation through complex mechanisms that vary with exposure duration and concentration. Chronic stress induces persistent hypothalamic–pituitary–adrenal (HPA) axis activation, resulting in sustained corticosterone elevation [79]. Prolonged glucocorticoid exposure leads to glucocorticoid receptor (GR) desensitization and downregulation, which paradoxically enhances NLRP3 inflammasome activation via dysregulated GR/NF-κB crosstalk, thereby promoting microglial polarization toward a pro-inflammatory phenotype [80]. Concurrently, cortisol-mediated hyperexcitation of glutamatergic neurons triggers excessive ATP release and damage-associated molecular pattern (DAMP) production. These signaling molecules, in combination with mitochondrial dysfunction-derived reactive oxygen species, potently reactive microglial purinergic receptors and Toll-like receptors, creating a self-amplifying cycle of neuroinflammation that disrupts hypothalamic homeostasis.

In addition, cortisol disrupts the integrity of the blood–brain barrier by down-regulating tight junction proteins, allowing peripheral inflammatory factors to infiltrate the central nervous system [102,103]. This induces central insulin resistance and epigenetic modifications of pro-inflammatory genes, such as DNA methylation alterations. These factors together constitute the microenvironment that induces sustained reactivation of microglia [81]. TNF-a released by reactive microglia promotes the internalization of α-amino-3-hydroxy-5-methyl-4-isoxazolepropionic acid (AMPA) receptors, significantly weakening synaptic transmission efficiency, which not only leads to anxiety-like behaviors but also alters sympathetic nerve tension and neuronal synaptic plasticity [68]. Meanwhile, IL-1β secreted by microglia specifically suppresses the discharge activity of paraventral nucleus PVN oxytocin neurons, thereby reducing social behavior and energy expenditure, forming a vicious cycle of neuroendocrine-immune-behavior [69].

### 2.3. Microglia Regulation by Remote Gut Microbiota and Microbial Toxins

Hormonal and lipid signals primarily originate from “direct” peripheral organs (adipose, adrenal glands) that have well-characterized anatomical connections to the hypothalamus. However, the gut microbiota—an often-overlooked peripheral “organ”—exerts indirect yet powerful control over hypothalamic microglia through a unique “gut–brain axis.” Unlike lipid metabolites or hormones that act as individual signaling molecules, the gut microbiota modulates microglia via a combination of microbial metabolites, microbial toxins, and neuroactive substances, creating a complex regulatory network that links gut health to hypothalamic function. Importantly, gut microbiota dysbiosis such as following HFD or chronic stress, can disrupt this balance, turning a protective microbial signal into a pro-inflammatory trigger for microglia. The subsequent section details how the gut microbiota and its derivatives regulate hypothalamic microglia, and how this crosstalk contributes to systemic homeostasis and disease.

The gut microbiota, often referred to as the “second genome” of the human body, plays an indispensable role in maintaining host homeostasis. It not only regulates energy metabolism and immune balance by fermenting dietary fibers to produce short-chain fatty acids (SCFAs) such as butyric acid and propionic acid, but also synthesizes neuroactive substances, such as 5-HT precursors and γ-aminobutyric acid (GABA), that directly influence brain function [82]. Furthermore, through a bidirectional communication system known as the “brain–gut axis,” it modulates both the gut nervous system and vagus nerve signaling, along with blood–brain barrier permeability and microglial cell activity [83,84,104]. This involvement is crucial in the development of metabolic diseases, mental disorders, and neurodegenerative conditions [83]. Short-chain fatty acids produced by gut microbiota fermentation of dietary fiber—particularly butyric acid and propionic acid—can traverse the blood–brain barrier into the hypothalamic region. These metabolites significantly inhibit NF-κB signaling pathway activity by binding to G protein-coupled receptors (GPR43/41) on microglial surfaces, thereby reducing the release of pro-inflammatory factors such as IL-6 and TNF-α [83]. Additionally, SCFAs have been shown to effectively maintain microglial homeostasis by suppressing histone deacetylase (HDAC) activity while upregulating anti-inflammatory gene expression including IL-10 [85,105]. However, when there is disruption in gut microbiota composition, increased intestinal permeability allows lipopolysaccharides (LPS) to enter circulation. This triggers activation of hypothalamic microglia via the CD14/TLR4 signaling pathway, consequently altering their regulatory functions related to neuronal homeostasis [86].

In certain pathological conditions, numerous small molecules can traverse the blood–brain barrier to directly reactive microglia in the hypothalamic region. Excessive alcohol consumption leads to the activation of the NADPH oxidase-ROS-NF-κB signaling axis via TLR4/2 receptors by both alcohol and its metabolites, thereby promoting the release of pro-inflammatory factors [87,106]. Neurotoxins trigger the assembly of NLRP3 inflammasomes through pattern recognition receptors, which drives microglial transformation into a pro-inflammatory phenotype [88]. Additionally, ATP activates pannexin-1 channels and NLRP3 inflammasomes through purinergic receptors (P2X7/P2Y12), facilitating the maturation and release of IL-1β [90,91]. These activation processes collectively enhance NF-κB and MAPK signaling pathways, resulting in morphological alterations in microglia characterized by reduced branching and increased cell size. This cascade culminates in a positive feedback loop of inflammation that disrupts glial interaction balance, ultimately leading to pathological changes such as impaired synaptic plasticity and dysfunction of the BBB.

The aforementioned peripheral stimuli—lipid metabolites, hormones, and gut microbiota derivatives—converge on hypothalamic microglia, triggering their activation through shared and distinct signaling pathways (e.g., TLR4/NF-κB, LepRb/STAT3, GPR43/HDAC). However, microglia do not merely “respond” to these signals; they act as central integration hubs that translate peripheral inputs into coordinated changes in hypothalamic function. This integration relies on three core mechanisms: modulating neuronal activity via inflammatory mediators, reshaping neural circuits through synaptic pruning, and remodeling the extracellular environment via phagocytosis. Together, these mechanisms enable microglia to bridge peripheral signals with hypothalamic neuro-immune-endocrine-metabolic output, ultimately governing systemic homeostasis. The following section delves into the mechanistic details of how microglia execute this central integration role.

## 3. Mechanistic Insights of Microglia as Central Hubs in Hypothalamic Neuro-Immune-Endocrine-Metabolic Integration

**Figure 2:**
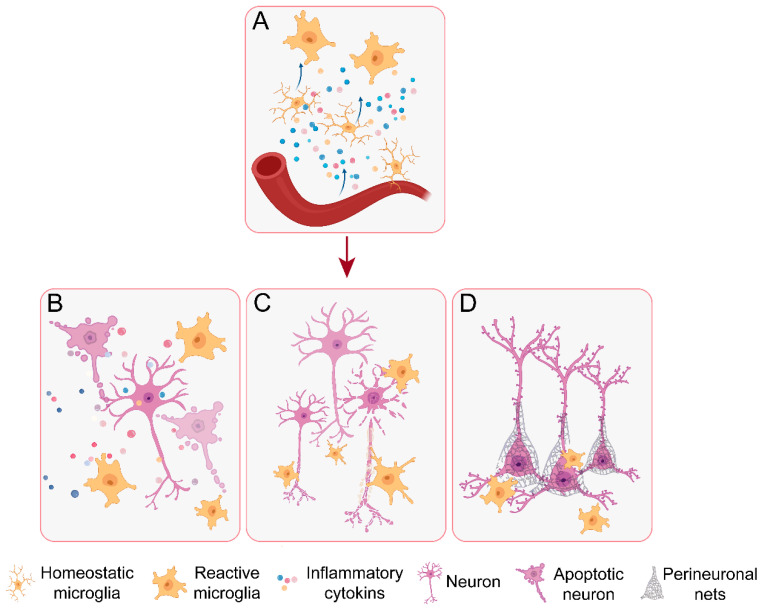
Mechanisms of microglia-neuron interactions in the hypothalamus: (**A**) Activation pathways: Peripheral organ-derived signals prime microglial activation through diverse stimuli. (**B**) Inflammatory cytokins modulation: Reactive microglia release inflammatory mediators that differentially regulate neuronal survival (promoting apoptosis or supporting neurodevelopment) and synaptic efficacy. (**C**) Circuit refinement: Microglia mediate activity-dependent synaptic pruning to optimize neural network connectivity. (**D**) Phagocytic regulation: Direct engulfment of neuronal components (including perineuronal nets) alters ionic homeostasis and synaptic plasticity through structural remodeling.

Microglia serve as pivotal immune regulators within the hypothalamus, orchestrating neuroimmune-endocrine-metabolic homeostasis through multifaceted mechanisms. Their unique perivascular localization renders them particularly sensitive to peripheral signals, enabling rapid activation and subsequent modulation of neuronal activity and neuroendocrine axis function via cytokine-mediated signaling. Beyond classical immune functions, microglia exhibit remarkable plasticity in shaping neural circuits through activity-dependent synaptic pruning. Recent advances have further revealed their capacity to directly remodel the neuronal microenvironment via phagocytic activity, thereby dynamically tuning neuronal sensitivity to metabolic cues. These discoveries fundamentally expand our understanding of neuro-immune crosstalk and provide novel mechanistic insights for developing targeted therapeutic interventions against metabolic and neurological disorders (Figure 2, Table 2).

### 3.1. Immunomodulatory Mechanisms of Hypothalamic Microglia

The paraventricular nucleus, while residing within the blood–brain barrier-protected brain region, displays unique vascular characteristics that distinguish it from other brain areas. Its capillary network features a complex architecture marked by smaller vessel diameters, extensive branching patterns, and reduced vascular straightness. This specialized microvascular geometry significantly increases the endothelial surface area, promoting enhanced cellular interactions with resident microglia and facilitating greater permeability to peripheral signals [115]. Consequently, hypothalamic microglia in this region exhibit heightened susceptibility to peripheral stimuli, readily transitioning from their surveillance state to a reactive phenotype (The reactive phenotype of microglia refers to the distinct functional and morphological states exhibited by microglia when they are activated from a quiescent state upon detecting disruptions to the homeostatic balance of the brain’s internal environment (such as injury, infection, disease, or exposure to toxins). This is not merely a simple “on/off” switch, but rather a complex, polymorphic continuous spectrum. As the resident macrophages of the central nervous system (CNS), it was natural for scientists to draw upon the well-established macrophage polarization model (i.e., the M1/M2 model) in immunology when initiating research on the activated states of microglia: The classical activated phenotype is primarily neurotoxic, analogous to the M1 phenotype of macrophages. This phenotype plays a crucial role in acute infections and pathogen clearance; however, excessive or sustained activation can exacerbate neuroinflammation. In contrast, the alternative activated phenotype resembles the M2 phenotype of macrophages and is mainly neuroprotective and restorative in nature. Its functions include limiting inflammatory damage, clearing “debris” (e.g., apoptotic cell fragments), promoting tissue healing, and supporting neuronal survival. Modern perspectives further emphasize that the reactive phenotype of microglia exists as a continuous spectrum, rather than discrete, mutually exclusive states. Microglia can dynamically switch between different functional states in response to changes in environmental signals.). Upon reactivation, these microglia predominantly release inflammatory mediators including TNF-α, IL-1β, IL-6, and reactive oxygen species (ROS), which collectively modulate neuronal excitability and synaptic plasticity [107].

Among these factors, TNF-α promotes apoptosis in hypothalamic neurons by activating the Caspase-3 pathway and influences the secretion of CRH and TRH, ultimately leading to dysfunction within the HPA axis [36]. Additionally, TNF-α can activate the NF-κB signaling pathway via its receptor TNFR1; this upregulates AMPA receptors—specifically GluA2-deficient subtypes—and enhances neuronal excitability. Such changes may result in abnormal discharges from hypothalamic neurons that disrupt appetite regulation mechanisms, such as leptin resistance. IL-1β can activate N-methyl-D-aspartic acid (NMDA) receptors through IL-1R, increase Ca^2+^ influx, induce neuronal overexcitation, and even excitotoxicity, affecting thermoregulation [109]. Additionally, it can also reduce brain-derived neurotrophic factor (BDNF) expression, impair synaptic formation in hypothalamic neurons, and affect neural circuits related to learning and memory [108]. IL-6 can suppress synaptic plasticity in hypothalamic arcuate nucleus neurons, reduce the activity of POMC neurons (appetite suppression), and promote the activation of AgRP neurons (appetite promotion), leading to metabolic disorders [67]. ROS and inducible nitric oxide synthase (iNOS) compromise mitochondrial function in hypothalamic neurons through oxidative stress, adversely affecting energy metabolism regulation—including leptin and insulin signaling pathways [64].

### 3.2. Activity-Dependent Synaptic Pruning by Microglia

The release of inflammatory mediators by activated microglia represents a “fast-acting” mechanism to modulate neuronal function, as cytokines and ROS can rapidly alter neuronal excitability and hormone secretion. However, for long-term adaptation of hypothalamic circuits to persistent peripheral signals, like chronic HFD and sustained stress, microglia rely on a more “structural” mechanism: activity-dependent synaptic pruning. This process allows microglia to selectively eliminate or strengthen synapses between key hypothalamic neurons, thereby reconfiguring neural circuits to match the current physiological state. Importantly, synaptic pruning is not independent of immune modulation—cytokines like TNF-α and IL-1β can also regulate synaptic strength, creating a synergy between “chemical” and “structural” mechanisms. The following section explores how microglia mediate synaptic pruning in the hypothalamus and its functional consequences for homeostasis.

Researchers have elucidated the critical role of microglia in dynamically shaping neural circuits through activity-dependent synaptic pruning, a process fundamental to neural plasticity and circuit homeostasis [72,73,116]. Under physiological conditions, this sophisticated pruning mechanism maintains cardiovascular homeostasis by fine-tuning the excitation-inhibition balance among PVN neurons. During acute stress responses, microglia rapidly amplify their pruning activity to enhance sympathetic drive, demonstrating their remarkable adaptability in coordinating systemic physiological adaptations. In the ARC, microglia maintain metabolic balance by precisely regulating synaptic connectivity between orexigenic AgRP neurons and anorexigenic POMC neurons [117,118]. Similarly, within the paraventricular nucleus, microglial-mediated synaptic remodeling serves as a key mechanism for autonomic nervous system regulation, influencing sympathetic outflow and associated neural pathways [119]. These findings underscore microglia as central regulators of neurocircuit plasticity, capable of dynamically adjusting their activity to meet both basal homeostatic demands and acute physiological challenges.

Microglia execute synaptic pruning through three principal molecular pathways: complement-dependent phagocytosis, cytokine-mediated synaptic modulation, and purinergic signaling. The complement cascade initiates synaptic clearance when microglial CR3 receptors recognize neuronal complement proteins (C1q/C3). Concurrently, microglia-secreted cytokines like TNF-α and IL-1β dynamically regulate synaptic strength and stability. Furthermore, ATP-mediated activation of microglial P2Y12 receptors facilitates their targeted migration and synaptic contact formation [72,90,92,120,121].

### 3.3. Extracellular Matrix Remodeling by Direct Microglial Phagocytosis

Synaptic pruning focuses on modifying the “functional connections” between neurons, but microglia’s role in reshaping hypothalamic circuits extends to the “structural scaffold” that surrounds neurons: the extracellular matrix (ECM). Perineuronal nets (PNNs)—a specialized component of the ECM—act as physical barriers that restrict synaptic plasticity, particularly in maturing neurons. By phagocytosing PNNs, microglia can “release” this structural constraint, enabling long-term changes in neuronal morphology and synaptic connectivity that go beyond the scope of synaptic pruning alone. This mechanism is especially critical for hypothalamic neurons involved in metabolic regulation and sexual differentiation, where ECM remodeling directly influences neuronal sensitivity to physiological cues. The subsequent section examines how microglial phagocytosis of the ECM modulates hypothalamic function and contributes to both physiological adaptation and pathological dysfunction.

Emerging evidence reveals that microglia extend their regulatory functions beyond classical neuroinflammation and synaptic pruning, playing a more sophisticated role in neural circuit plasticity through dynamic structural remodeling [106]. A key mechanism involves their phagocytic activity toward PNNs—specialized extracellular matrix structures enriched in chondroitin sulfate proteoglycans that predominantly envelop inhibitory neurons like AgRP neurons [111,112]. PNN formation coincides with neuronal maturation, with studies demonstrating significant accumulation in the ARC beginning at postnatal day 12 (P12) and persisting into adulthood. Functionally, PNNs serve as physical constraints on synaptic plasticity. Microglia-mediated PNN clearance creates a permissive microenvironment that facilitates AgRP neuronal synaptic growth, whereas excessive PNN accumulation may prematurely stabilize neuronal morphology and restrict developmental plasticity. Notably, leptin signaling—a crucial nutritional cue for AgRP neuron maturation—shows enhanced neuronal responsiveness following microglial PNN phagocytosis, evidenced by increased pSTAT3 expression, independent of circulating leptin levels. These discoveries highlight how microglial regulation of the extracellular matrix directly modulates neuronal intrinsic sensitivity to metabolic signals.

New evidence demonstrates that HFD consumption elevates prostaglandin E2 (PGE2) levels in the mediobasal hypothalamus, where it activates EP4 receptors on microglia to modulate their phagocytic activity. Genetic ablation of microglial EP4 results in a functionally impaired phagocytic phenotype, characterized by reduced CD68 expression and diminished physical interactions with POMC neuronal processes. These cellular alterations are associated with enhanced POMC neurite density projecting to the PVN, suggesting that EP4-mediated microglial activity normally restricts POMC axonal arborization. Importantly, this microglia-dependent circuit remodeling mechanism contributes to the development of HFD-induced metabolic dysfunction, as evidenced by attenuated weight gain and improved insulin sensitivity in microglial EP4 knock-out mice. These findings establish microglial EP4 signaling as a critical molecular switch that translates dietary lipid overload into neuroinflammatory responses, ultimately promoting metabolic imbalance through structural plasticity of anorexigenic POMC circuits [113].

The sexually dimorphic nucleus of the preoptic area (SDN-POA) represents one of the most pronounced and evolutionarily conserved sex differences in the mammalian brain. Emerging evidence has revealed that this structural dimorphism is critically regulated by microglia-mediated phagocytic activity, with female SDN exhibiting significantly greater microglia-dependent neuronal engulfment that actively promotes neuronal elimination during development. Experimental studies demonstrate that selective inhibition of microglial phagocytic function transiently prevents neuronal apoptosis and increases SDN volume in hormonally naive females, establishing a novel mechanistic framework for understanding the neuroimmune basis of sexual differentiation [114]. These findings provide compelling evidence that microglial activity serves as a key determinant of SDN sexual dimorphism independent of classical hormonal regulation.

## 4. Targets and Regulatory Functions of Hypothalamic Microglia as Orchestrator of the Center-Periphery Axis

**Figure 3:**
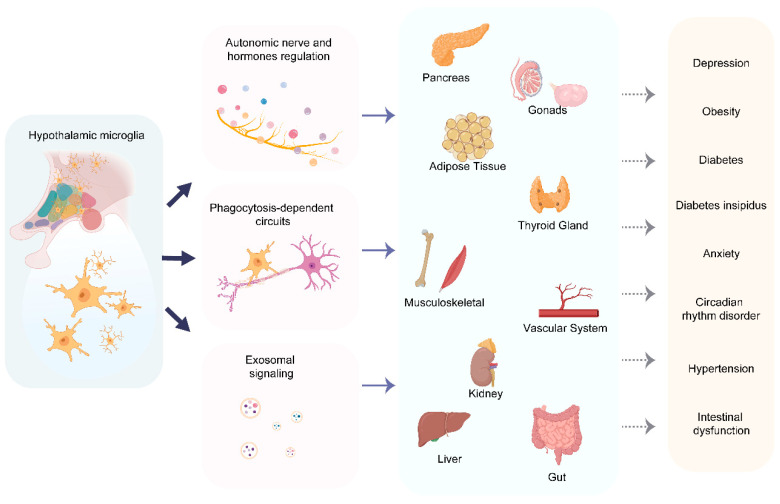
Hypothalamic microglial regulation of peripheral organs and the associated disorders The reactive microglia in hypothalamus disrupt peripheral organ function through: (i) cytokine-mediated dysregulation of autonomic nervous system and hypothalamic–pituitary–adrenal (HPA) axis activity; (ii) phagocytosis-dependent remodeling of hypothalamic nuclei and neural circuits; and (iii) exosome-mediated delivery of neuroinflammatory signals to peripheral tissues (including liver, adrenal glands, vasculature, gastrointestinal tract, thyroid, gonads, adipose tissue, and pancreas). These multidirectional communication pathways collectively contribute to the development of hypothalamic microglia-associated disorders.

In health, hypothalamic microglia serve not merely as passive recipients of peripheral signals, but as active effectors that orchestrate central regulation of peripheral organ function. Through their integrative role in the neuro-immune-endocrine axis, reactive microglia dynamically modulate metabolic, immune, and autonomic functions, thereby establishing a sophisticated brain–periphery feedback loop. These specialized immune cells exert precise control over peripheral systems via three principal efferent pathways: autonomic regulation through sympathetic/parasympathetic modulation of cardiovascular activity, insulin secretion, and hepatic glucose output; neuroendocrine coordination via the HPA axis to influence corticosterone and insulin levels; and immune–metabolic coupling through exosome-mediated inter-organ communication that can either exacerbate or ameliorate peripheral inflammation (Figure 3, Table 3). When microglial function becomes dysregulated in pathological states, this intricate regulatory network is disrupted, leading to a constellation of systemic disorders including circadian rhythm abnormalities, water-electrolyte imbalance, hypertension, insulin resistance, anxiety-depression behaviors, and obesity—all manifestations of impaired brain–periphery communication.

Among the three efferent pathways of hypothalamic microglia, the autonomic nervous system (ANS) acts as the “fast-response channel” for peripheral regulation. This is because sympathetic/parasympathetic signals can directly innervate peripheral organs, such as the heart, pancreas, gut, within seconds to minutes, enabling rapid adaptation to acute physiological changes. Notably, the ANS regulation by microglia is not a standalone process—it relies on the central integration mechanisms detailed earlier to “translate” peripheral stimuli into precise autonomic outputs. The following section focuses on how microglia tune parasympathetic and sympathetic activity to control visceral function and peripheral organ homeostasis, along with the pathological consequences of dysregulated ANS control.

### 4.1. Autonomic Nervous System Regulation

The parasympathetic branch of the ANS, primarily mediated by the vagus nerve, is critical for “rest-and-digest” processes—including gastrointestinal motility, pancreatic insulin secretion, and visceral homeostasis. Hypothalamic microglia modulate parasympathetic activity by targeting key neural circuits (hypothalamic-vagal pathways) and releasing soluble factors that fine-tune vagal efferent signaling. This regulation is particularly evident in metabolic and gastrointestinal contexts, where microglia-derived cytokines act as “neuro-modulators” to link central inflammation with peripheral visceral function.

#### 4.1.1. Parasympathetic Modulation of Visceral Function

The cephalic phase of insulin secretion is primarily mediated through vagal nerve activation rather than direct glucose stimulation of pancreatic β cells. Recent studies have identified IL-1β as a novel regulator of postprandial insulin release, with microglia-derived IL-1β demonstrating incretin-like properties through neuronal transmission pathways. Specifically, IL-1β enhances vagal efferent activity to stimulate insulin secretion while simultaneously modulating hypothalamic responsiveness to cephalic stimuli [110].

Chronic microglial reactivation leads to excessive release of pro-inflammatory cytokines that dysregulate vagal signaling pathways, resulting in altered gastrointestinal motility patterns and subsequent changes in gut microbiota composition [122,123,126,139,140]. These pathological alterations establish a self-perpetuating cycle that contributes to the development of visceral hypersensitivity and various gastrointestinal disorders, including functional dyspepsia and irritable bowel syndrome.

While the parasympathetic system maintains basal visceral homeostasis, the sympathetic branch dominates “fight-or-flight” responses—governing cardiovascular function, hepatic glucose output, and peripheral metabolism. In contrast to the parasympathetic system’s reliance on vagal signaling, sympathetic regulation by microglia often targets the PVN (a core presympathetic nucleus), where microglial activation disrupts the excitation-inhibition balance of presympathetic neurons. This dysregulation is particularly prominent in cardiovascular and metabolic diseases, where a “sympathetic overdrive” driven by microglia becomes a key pathological feature.

#### 4.1.2. Sympathetic Activation in Regulating Peripheral Organ Functions

In hypertension, hemodynamic disturbances in the PVN microvasculature, including elevated blood pressure and altered shear stress, promote increased vascular ATP leakage into the brain parenchyma. This extracellular ATP activates microglial P2Y12 receptors, triggering C/EBPβ-dependent upregulation of proinflammatory mediators that directly enhance presympathetic neuronal excitability while suppressing parasympathetic activity [90,127,128,129]. This ensuing sympathetic overdrive manifests as vasoconstriction, tachycardia, and activation of the renin-angiotensin system. Collectively, these responses establish a self-sustaining cycle wherein hypertension induces ATP leakage, subsequently reactivating microglia. Microglial reactivation then reinforces sympathetic hyperactivity, resulting in progressively worsening hypertension and ultimately culminating in impaired cardiovascular function.

Complementary studies reveal context-specific PVN dysregulation across pathological states. In myocardial infarction models, METTL3-mediated m6A modification within microglia activates the TRAF6/ECSIT signaling pathway, induces mitochondrial oxidative stress in PVN neurons, and stimulates sympathetic hyperactivity, thereby contributing to the development of post-MI ventricular arrhythmias [130,141]. Following traumatic brain injury (TBI), neutrophil extracellular traps (NETs) in the PVN stimulate microglia to secrete inflammatory cytokines through HMGB1/JNK/AP1 signaling, exacerbating sympathetic hyperactivity and poor prognosis [142].

### 4.2. Neuroendocrine Signaling Networks

The autonomic nervous system provides rapid, short-term regulation of peripheral organs, but long-term systemic homeostasis requires the “slow-acting yet sustained” control of the neuroendocrine system. Hypothalamic microglia bridge these two systems by coordinating the ANS with neuroendocrine axes—most notably the HPA axis and hypothalamic-pancreatic neural pathways. For example, microglia that activate sympathetic hyperactivity in hypertension also enhance HPA axis activity to sustain corticosterone release, creating a “synergistic loop” of stress responses. The following section explores how microglia modulate neuroendocrine signaling networks to govern metabolic homeostasis and pancreatic β-cell function, and how this regulation goes awry in disease.

The HPA axis is the primary neuroendocrine pathway for stress adaptation and metabolic regulation, linking hypothalamic CRH neurons to pituitary-adrenal hormone release. Hypothalamic microglia act as “gatekeepers” of HPA axis activity: under physiological stress, they fine-tune CRH neuron excitability to maintain glucocorticoid homeostasis; under pathological conditions (e.g., chronic stress, obesity), they drive HPA axis hyperactivation or hypoactivity, leading to systemic metabolic dysfunction. This bidirectional regulation relies on microglial-derived cytokines (e.g., TNF-α, IL-1β) and synaptic remodeling—mechanisms that directly connect central inflammation to neuroendocrine output.

#### 4.2.1. HPA Axis Dynamics in Regulating Metabolic Homeostasis

Under stress conditions, reactive microglia enhance the excitability of CRH neurons through neuroinflammatory signaling and synaptic remodeling, leading to hyperactivation of the HPA axis and subsequent elevation of glucocorticoid levels. This neuroendocrine alteration exerts profound effects on peripheral metabolic organs, where increased corticosterone stimulates adipose tissue lipolysis via hormone-sensitive lipase (HSL) activation, elevating circulating free fatty acids, while concurrently suppressing glucose transporter expression in adipocytes to impair insulin-dependent glucose uptake, ultimately contributing to systemic insulin resistance [143,144,145,146]. Intriguingly, in chronic stress models, inhibition of microglial activation and NLRP3-mediated inflammatory pathways has been shown to mitigate stress-induced bone loss and osteoporosis [80]. Conversely, under hypoglycemic conditions, aberrant microglial activation interacts with orexigenic neuropeptide Y (NPY) neurons to suppress HPA axis activity, and microglial inhibition enhances counterregulatory responses to both insulin-induced and neurogenic hypoglycemia [131]. Collectively, these findings unveil novel molecular mechanisms underlying the intricate interplay among neural, immune, and endocrine systems, demonstrating how central inflammatory processes dynamically modulate peripheral metabolic homeostasis through bidirectional regulation of the HPA axis in response to diverse metabolic challenges.

#### 4.2.2. Hypothalamic Microglia-Neural Control of Pancreatic β Cells

While the HPA axis regulates metabolic homeostasis through systemic hormone release, the hypothalamus also exerts “direct neural control” over peripheral metabolic organs—most notably the pancreas. This control is mediated by hypothalamic neurons that project directly to pancreatic β cells, and microglia act as “modulators” of this neural pathway. In metabolic diseases like obesity, microglial dysfunction disrupts the inhibitory/excitatory balance of AgRP-pancreatic neural projections, leading to abnormal insulin secretion—an effect that complements the metabolic dysregulation caused by HPA axis dysfunction.

Emerging evidence suggests that microglia-mediated hypothalamic inflammation is a driver of metabolic dysfunction, particularly insulin resistance and type 2 diabetes [110,124,125]. The study found that hypothalamic AgRP neurons directly regulate pancreatic β-cell function through neural projections, a process finely regulated by microglia [125]. AgRP neurons release gamma-aminobutyric acid through their axonal terminals, exerting direct inhibitory regulation on pancreatic β cells. In the state of metabolic disorder induced by a high-fat diet, reactive microglia significantly reduced the inhibitory input of AgRP neurons to β cells by secreting TGF-β, thereby promoting pulsatile insulin secretion [132,147]. This neuro-immune-endocrine interaction mechanism reveals new pathways by which the central nervous system regulates through direct neural projections and peripheral metabolic organ functions, providing an important theoretical basis for understanding the pathogenesis of abnormal insulin secretion in metabolic diseases.

### 4.3. Immune–Metabolic Crosstalk via Exosomal Signaling

The autonomic and neuroendocrine pathways rely on “direct” signaling to regulate peripheral organs, but hypothalamic microglia also employ “indirect, long-distance” communication via exosomes—nanoparticles that encapsulate bioactive molecules such as miRNAs, cytokines and circulate systemically. Exosomal signaling is particularly critical in chronic pathological states, where it enables microglia to exert sustained effects on distant organs and amplify neuroinflammation-peripheral dysfunction loops. Unlike the rapid effects of the ANS or the sustained effects of the HPA axis, exosomal regulation is “targeted” (via organ-specific uptake) and “multifaceted” (via diverse cargo molecules), adding a new layer of complexity to microglia’s role in center-periphery axis control.

Emerging evidence highlights hypothalamic microglia as pivotal neuroimmune regulators that exert remote control over peripheral organs through the release of specialized exosomes [148,149,150]. Reactive microglia-derived exosomes encapsulate diverse bioactive molecules, with mechanistic studies revealing their bidirectional pathological impacts. Notably, exosomal MCP1 elevation triggers MCP1/CCR2 signaling to accelerate β-endorphin neuronal apoptosis, thereby exacerbating neuroendocrine and behavioral stress responses [87]. Alcohol-reactive hypothalamic microglia release miR-155-enriched exosomes that circulate systemically, aggravating hepatic injury through hepatic infiltration while concurrently worsening acute kidney injury via renal accumulation [89,133]. These exosomes further impair adipogenesis in preadipocytes and promote white adipose tissue browning, establishing a vicious cycle where peripheral inflammatory factors reciprocally cross the blood–brain barrier to reactivate central microglia. This maladaptive crosstalk perpetuates neuroinflammation and metabolic dysregulation. Intriguingly, miR-124-3p enhances thermogenesis by elevating mitochondrial content in brown adipocytes, while intracellular succinate accumulation amplifies mitochondrial complex II activity, suggesting compensatory energy regulation mechanisms during metabolic stress [151].

The preceding sections detail how hypothalamic microglia regulate peripheral organs via the ANS, HPA axis, and exosomal signaling—but when these regulatory mechanisms are disrupted, they converge to drive a spectrum of systemic diseases. These diseases, though clinically distinct, share a common pathological root: hypothalamic microglia dysfunction. Below is an integration of key disease phenotypes and their links to microglial dysregulation, along with potential clinical implications.

Hypothalamic microglial activation induced by an HFD—primarily mediated through the TLR4/NLRP3 signaling pathway—serves as a pivotal driver of metabolic dysregulation by disrupting three core regulatory cascades. First, activated microglia trigger sympathetic overdrive, which enhances hepatic gluconeogenesis and promotes lipolysis in adipose tissue; these effects collectively exacerbate systemic metabolic imbalance by increasing circulating glucose and free fatty acid levels. Second, microglial activation leads to hyperactivation of the HPA, resulting in elevated corticosterone secretion. Sustained high corticosterone levels further aggravate insulin resistance in peripheral tissues such as adipocytes, skeletal muscle, as this hormone suppresses insulin-dependent glucose transporter expression and activity. Third, HFD-activated microglia release exosomes enriched with miR-155; upon systemic circulation, this microRNA impairs adipocyte differentiation and compromises pancreatic β-cell function. Additionally, microglia mediate aberrant pruning of inhibitory synapses on AgRP neurons, reducing the release of GABA onto pancreatic β-cells. This loss of inhibitory GABAergic input disrupts the precise temporal and amplitude control of insulin secretion, contributing to further metabolic dysregulation [64,89,125].

Hypothalamic microglial dysfunction plays a critical role in the progression of cardiovascular diseases by disrupting the “neuroinflammation-sympathetic” regulatory axis, with distinct mechanisms observed in hypertension and post-myocardial infarction states. In hypertension, hemodynamic disturbances trigger ATP leakage from the microvasculature of the hypothalamic PVN. Extracellular ATP activates microglial P2Y12 receptors, initiating a signaling cascade that drives C/EBPβ-dependent transcription of proinflammatory cytokines. These cytokines directly enhance the excitability of PVN presympathetic neurons, leading to sympathetic overdrive characterized by vasoconstriction, tachycardia, and activation of the renin-angiotensin system [90,127].

Following MI, a distinct microglial-mediated mechanism contributes to cardiovascular dysfunction, METTL3-dependent m^6^A methylation in PVN microglia. This epigenetic modification enhances the stability of transcripts encoding TRAF6 and ECSIT—key regulators of mitochondrial oxidative stress—leading to excessive ROS production in microglia. ROS released by activated microglia diffuses to adjacent PVN neurons, inducing mitochondrial dysfunction and further increasing neuronal excitability. This cascade exacerbates sympathetic hyperactivity, which in turn increases the risk of ventricular arrhythmias and impairs post-MI cardiac remodeling [130].

Hypothalamic microglial reactivation serves as a common pathogenic link between neuropsychiatric symptoms such as anxiety, depression, and multiorgan injury, with context-specific mechanisms in chronic stress and alcohol exposure. Under chronic stress, dysregulation of the GR/NF-κB signaling pathway drives hypothalamic microglial activation: sustained corticosterone exposure desensitizes GRs in microglia, disrupting the inhibitory effect of GR on NF-κB and leading to excessive proinflammatory cytokine production. Microglia-derived IL-1β suppresses the firing activity of PVN oxytocin neurons, a key cell population regulating social behavior and stress resilience, contributing to social dysfunction. And microglia-secreted TNF-α impairs the trafficking of AMPA receptors to neuronal membranes, reducing synaptic transmission efficiency in circuits mediating mood regulation and ultimately leading to anxiety-like behaviors [68,69]. As a central hub for emotional regulation, the hypothalamus achieves homeostatic control of emotions by forming neural circuits with nuclei including the amygdala, hippocampus, and prefrontal cortex. Dysfunction of microglia leads to impaired synaptic pruning, which in turn contributes to the development of neuropsychiatric and cognitive-related disorders.

In the context of alcohol exposure, hypothalamic microglia mediate a “brain–periphery–brain” loop of multiorgan injury. Alcohol and its metabolites, like acetaldehyde, activate microglia via TLR4/2 signaling, prompting the release of exosomes enriched with miR-155. These exosomes target peripheral organs: in the liver, miR-155 suppresses SIRT1, exacerbating alcohol-induced liver injury; in the kidneys, miR-155 promotes oxidative stress by downregulating antioxidant genes, contributing to renal dysfunction. Concurrently, alcohol-induced gut dysbiosis increases intestinal permeability, allowing LPS to enter the systemic circulation. Peripheral LPS crosses the BBB and further reactivates hypothalamic microglia, perpetuating the cycle of neuroinflammation and multiorgan injury [86,89].

The integration of these disease phenotypes reveals a unifying principle: hypothalamic microglia serve as “central nodes” governing systemic homeostasis, and their dysfunction represents a shared pathogenic driver across metabolic, cardiovascular, and neuropsychiatric disorders. Moving forward, clinical strategies could prioritize three interconnected directions: first, exploring targeted delivery systems, such as PVN-directed nanoparticles, to deliver microglia modulators (including EP4 antagonists and TLR4 inhibitors), ensuring precise action on hypothalamic microglia while minimizing off-target effects. Second, advancing the development of disease-specific biomarkers, with circulating microglia-derived exosomal miR-155 standing out as a potential candidate to predict the severity of alcohol-related organ injury, enabling early intervention before irreversible damage occurs. Third, designing combination therapeutic regimens that pair peripheral interventions, like insulin sensitizers for metabolic disorders, with central anti-inflammatory agents, a strategy aimed at breaking the self-perpetuating “brain–periphery” vicious cycles underlying many chronic diseases. By centering hypothalamic microglia as a therapeutic target, this approach holds promise for developing “cross-disease” treatments that address conditions traditionally managed as isolated entities, ultimately offering a more holistic and efficient approach to mitigating the global burden of multifactorial disorders.

### 4.4. A Paradigm of Microglial Dysregulation: The Case of Long COVID

Long COVID is a multisystem syndrome, and its core neurological symptoms (brain fog, fatigue, autonomic dysfunction)highly align with hypothalamic dysfunction [136]. A prominent hypothesis in the current scientific community posits that microglia-mediated chronic neuroinflammation is the core mechanism underlying a series of neurological symptoms (e.g., brain fog, fatigue) in Long COVID [152]. Clinical imaging studies have directly detected signs of microglial activation in the brains of patients with Long COVID [137]. The core pathway is hypothesized as follows: COVID-19 infection may act as an initial triggering event; even after the virus has been cleared, it may leave a lasting “inflammatory imprint” in the body, causing microglia to enter a persistent, dysfunctional reactive state. One of the key features of this state is downregulation of the P2Y12 receptor on the microglial surface—a receptor crucial for their normal environmental surveillance and repair functions. Loss of the P2Y12 receptor leads to microglial dysfunction, which in turn triggers neurovascular unit damage (including disruption of the blood–brain barrier) and initiates a cascading cycle of “glia-vascular-neuron” damage [138]. Dysfunctional microglia may also perform abnormal synaptic pruning and impair astrocyte-mediated support for neurons; together, these processes compromise neuroplasticity, providing a model to explain the physiological basis of “brain fog”.

Against this broader backdrop of neuroinflammation, hypothalamic microglia are thought to play an especially critical role. As a high-level command center regulating the autonomic nervous system, metabolism, sleep, and stress responses, hypothalamic dysfunction can perfectly account for the multisystem symptoms of Long COVID. Due to the unique properties of the blood–brain barrier in the hypothalamic region, this area is more vulnerable to the impact of peripheral inflammatory signals. When microglia within the hypothalamus are persistently activated, the local inflammatory factors they release directly disrupt the core neuronal circuits that regulate appetite, energy metabolism, sleep–wake cycles, and autonomic function. This can lead to metabolic issues, sleep disorders, abnormal heart rate, and other symptoms. Therefore, the hypothalamus is likely to be a key hub connecting neuroinflammation in the brain to systemic symptoms; its dysfunction creates a self-amplifying vicious cycle that sustains the complex manifestations of Long COVID. Based on this, regulating the harmful activity of microglia to shift them from a destructive inflammatory state to a reparative one is emerging as a highly promising new strategic direction for the treatment of Long COVID.

## 5. Conclusions and Outlook

The hypothalamus serves as the master regulator of systemic homeostasis, integrating various central and peripheral signals through its specialized neural architecture, including neurovascular units and glial networks. Within this critical hub, microglia have emerged as pivotal orchestrators of the neuro-immune-endocrine-metabolic network—transcending their conventional role as sentinels of neuroinflammation to exhibit sophisticated multidimensional regulatory capacities. This paradigm shift underscores their ability to coordinate complex physiological responses via multimodal signaling pathways involving purinergic receptors, chemokine gradients, and mitochondrial transfer mechanisms. Given their central positioning, microglial dysregulation represents a convergent pathological driver across diseases with high therapeutic resistance.

Future research should prioritize elucidating cell-specific microglial mechanisms—particularly their temporal-spatial activation patterns and metabolic reprogramming—in clinically intractable conditions. Key targets include oncological cachexia-associated hypothalamic inflammation, metabolic dysregulation in diabesity, sympathetic overdrive in heart failure, and neurovascular unit disruption post-stroke. Investigations must adopt the “periphery-hypothalamus-periphery” regulatory axis as an integrative framework, decoding how peripheral insults trigger microglial epigenetic remodeling in discrete hypothalamic nuclei, which subsequently amplifies systemic pathophysiology through neuroendocrine-autonomic efferent.

To clarify the dynamic regulatory mechanisms of microglia, future investigations must integrate multi-omics technologies, such as single-cell transcriptomics, spatial transcriptomics, and proteomics, to systematically characterize the spatiotemporal heterogeneity of microglial populations across different brain regions in both physiological and pathological contexts. Additionally, a holistic organismal perspective is crucial for analyzing multi-organ systems as integrated entities during concurrent or sequential pathologies. Through multilevel systems analysis, we can identify the dynamic behavioral patterns of microglia across various states, ultimately highlighting their central role within the neuroimmune regulatory network.

Complementing these efforts, the development of novel experimental models that faithfully recapitulate the unique microenvironmental features of the hypothalamus will provide robust platforms for deeper mechanistic understanding of microglial regulation. These integrated approaches will significantly advance the field of neuro-immune-endocrine-metabolic interactions, offering novel theoretical frameworks and therapeutic strategies for the diagnosis and treatment of related disorders.

Collectively, these efforts will propel the field of neuro-immune-endocrine-metabolic integration, offering transformative insights for diagnosing and treating associated diseases. By decoding the hypothalamus-periphery axis, we may uncover unified therapeutic strategies for conditions currently viewed as clinically distinct.

## Figures and Tables

**Table 1:** Central reactivation of hypothalamic microglia by peripheral afferents.

Key Mechanisms	Signaling Pathways	Disease Associations	References
Diet-induced Neuroinflammation	TLR4, NF-κB, NLRP3	Metabolic dysregulation, obesity, diabetes	[57,58,59,60,61,62,63,64]
Activation of Microglia and Cytokine Release	IL-1β, TNF-α, IL-6	Leptin resistance, increased appetite	[56,65,66,67,68,69]
Synaptic Remodeling and Pruning	IKKβ/NF-κB, JAK2/STAT3	Energy balance disruption, neuropsychiatric disorders	[64,70,71,72,73]
Leptin Regulation of Microglia	LepRb/JAK2/STAT3, PI3K/Akt	Leptin-related neuroinflammation, metabolic syndrome	[70,74,75,76,77,78]
Glucocorticoid Regulation of Microglia	GR/NF-κB, NLRP3	Chronic stress, anxiety disorders	[68,69,79,80,81]
Gut Microbiota Influence on Microglia	GPR43/41, CD14/TLR4	Metabolic diseases, neurodegenerative diseases	[82,83,84,85,86]
Anti-inflammatory Effects of Short-chain Fatty Acids	NF-κB, HDAC	Obesity, gut dysbiosis	[83,84]
Alcohol and Neurotoxin Activation of Microglia	TLR4/2, NADPH oxidase-ROS-NF-κB	Alcohol-related diseases, neurotoxic damage	[87,88,89]
Role of ATP in Microglial Activation	P2X7/P2Y12, NLRP3	Neuroinflammation, synaptic plasticity impairment	[90,91,92]

**Table 2:** Microglia–neuron crosstalk in hypothalamic circuits.

Key Mechanisms	Signaling Pathways	Disease Associations	References
Reactivity Shift in Microglia	TNF-α, IL-1β, IL-6, ROS	Neuroinflammation, metabolic dysregulation	[56,107,108]
Effects of TNF-α on Hypothalamic Neurons	Caspase-3, NF-κB, AMPA receptors	HPA axis dysfunction, appetite regulation disruption	[36,65,68]
Effects of IL-1β on Neurons	NMDA receptors, Ca^2+^ channels	Thermoregulation disorders, learning and memory impairments	[108,109,110]
Inhibitory Effects of IL-6 on Hypothalamic Neurons	POMC neurons, AgRP neurons	Metabolic syndrome, appetite disorders	[67,98]
Activity-dependent Synaptic Pruning	TNF-α, IL-1β, ATP/P2Y12	Neural circuit remodeling, metabolic balance disruption	[72,73,92]
Extracellular Matrix Remodeling and Microglial Phagocytosis	PNNs, phagocytic mechanisms	Neural plasticity, metabolic regulation disorders	[111,112,113]
Dietary Lipid Overload Effects on Microglia	PGE2, EP4 receptors	HFD-induced metabolic dysfunction	[113]
Sexual Dimorphism in Microglial Functions	Microglial phagocytosis	Sexual differences, neurodevelopmental disorders	[114]

**Table 3:** Hypothalamic microglia along the center–periphery axis: regulatory targets, efferent pathways, and systemic outcomes.

Key Mechanisms	Signaling Pathways	Disease Associations	References
Vagal Nerve-mediated Parasympathetic Regulation	IL-1β, neural transmission pathways	Gastrointestinal dysfunction, metabolic syndrome	[110,122,123]
Microglial Regulation of Insulin Secretion	IL-1β, vagal nerve activation	Diabetes, obesity	[110,124,125]
Chronic Microglial Activation and Gastrointestinal Motility	Pro-inflammatory cytokines	Functional dyspepsia, irritable bowel syndrome	[123,126]
Sympathetic Activation and Microglial Function	P2Y12 receptors, C/EBPβ	Hypertension, cardiovascular diseases	[90,127,128]
Microglial Role in Hypertension	ATP leakage, C/EBPβ-dependent pro-inflammatory factors	Cardiovascular dysfunction	[90,127,128,129]
Microglial Mediated Mechanisms Post-Myocardial Infarction	METTL3, TRAF6/ECSIT signaling pathways	Arrhythmias	[30,130]
HPA Axis in Metabolic Homeostasis Regulation	CRH, TNF-α, IL-1β	Stress response, metabolic dysregulation	[36,80,131]
Microglial Neural Control of Pancreatic β Cells	AgRP neurons, TGF-β	Type 2 diabetes, insulin resistance	[124,125,132]
Exosomal Signaling and Immune–Metabolic Crosstalk	miR-155, MCP1/CCR2	Multi-organ injury, alcohol-related diseases	[87,89,133]
Long COVID and Microglial Dysfunction	Downregulation of P2Y12 receptors, neuro-vascular-neuron damage	Brain fog, fatigue, autonomic dysfunction	[134,135,136,137,138]

## Data Availability

No datasets were generated or analyzed during the current study.

## Abbreviations

CNS	central nervous system
CRH	corticotropin-releasing hormone
TRH	thyrotropin-releasing hormone
GnRH	gonadotropin-releasing hormone
BBB	blood–brain barrier
PVN	paraventricular nucleus
MCP-1	monocyte chemoattractant protein-1
PRRs	pattern recognition receptors
HFD	high-fat diet
CVOs	circumventricular organs
ME	median eminence
PA	palmitate
POMC	pro-opiomelanocortin
AgRP	agouti-related peptide
HVZ	ventricular zone
LepRb	long-form leptin receptor
ARC	arcuate nucleus
HPA	hypothalamic–pituitary–adrenal
GR	glucocorticoid receptor
DAMP	damage-associated molecular pattern
AMPA	α-amino-3-hydroxy-5-methyl-4-isoxazolepropionic acid
SCFAs	short-chain fatty acids
GABA	γ-aminobutyric acid
HDAC	histone deacetylase
LPS	lipopolysaccharides
ROS	reactive oxygen species
NMDA	N-methyl-D-aspartic acid
BDNF	brain-derived neurotrophic factor
PNNs	perineuronal nets
PGE2	prostaglandin E2
SDN	sexually dimorphic nucleus
POA	preoptic area
TBI	traumatic brain injury
NETs	neutrophil extracellular traps
HSL	hormone-sensitive lipase
NPY	neuropeptide Y
